# The Origin, Development and Molecular Diversity of Rodent Olfactory Bulb Glutamatergic Neurons Distinguished by Expression of Transcription Factor NeuroD1

**DOI:** 10.1371/journal.pone.0128035

**Published:** 2015-06-01

**Authors:** Laurent Roybon, Teresa L. Mastracci, Joyce Li, Simon R. W. Stott, Andrew B. Leiter, Lori Sussel, Patrik Brundin, Jia-Yi Li

**Affiliations:** 1 Stem Cell laboratory for CNS Disease Modeling, Wallenberg Neuroscience Center, Department of Experimental Medical Science, BMC A10, Lund University, 22184 Lund, Sweden; 2 Strategic Research Area MultiPark, Lund University, 22184 Lund, Sweden; 3 Lund Stem Cell Center, Lund University, 22184 Lund, Sweden; 4 Department of Genetics and Development, Columbia University, 1150 St. Nicholas Ave., New York, New York, United States of America; 5 Department of Medicine, University of Massachusetts Medical School, Worcester, Massachusetts, United States of America; 6 John van Geest Centre for Brain Repair, University of Cambridge, Forvie Site, Robinson Way, Cambridge CB2 0PY, United Kingdom; 7 Van Andel Research Institute, Center for Neurodegenerative Science, 333 Bostwick Avenue NE, Grand Rapids, Michigan 49503, United States of America; 8 Neural Plasticity and Repair Unit, Wallenberg Neuroscience Center, Department of Experimental Medical Science, Lund University, BMC A10, 22184, Lund, Sweden; School of Biomedical Sciences, The University of Queensland, AUSTRALIA

## Abstract

Production of olfactory bulb neurons occurs continuously in the rodent brain. Little is known, however, about cellular diversity in the glutamatergic neuron subpopulation. In the central nervous system, the basic helix-loop-helix transcription factor NeuroD1 (ND1) is commonly associated with glutamatergic neuron development. In this study, we utilized ND1 to identify the different subpopulations of olfactory bulb glutamategic neurons and their progenitors, both in the embryo and postnatally. Using knock-in mice, transgenic mice and retroviral transgene delivery, we demonstrate the existence of several different populations of glutamatergic olfactory bulb neurons, the progenitors of which are ND1^+^ and ND1^-^ lineage-restricted, and are temporally and regionally separated. We show that the first olfactory bulb glutamatergic neurons produced – the mitral cells – can be divided into molecularly diverse subpopulations. Our findings illustrate the complexity of neuronal diversity in the olfactory bulb and that seemingly homogenous neuronal populations can consist of multiple subpopulations with unique molecular signatures of transcription factors and expressing neuronal subtype-specific markers.

## Introduction

The olfactory bulb (OB) contains granule and periglomerular interneurons, which are continually produced in the subventricular zone (SVZ) and migrate to the OB, forming the rostral migratory stream (RMS) in rodents [1, 2]. The OB also contains mitral and tufted cells, which originate in the rostral telencephalic buds and are the first glutamatergic neurons born during development [3–5]. While granule neurons are uniquely GABAergic, those reaching the OB to form the glomerular layer acquire distinct fates, depending on which transcription factors they express [6].

Until recently, the glutamatergic neurons that populate the OB were thought to be born exclusively during early embryogenesis. Recent findings, however, have shown that numerous migrating dorsal SVZ-derived neuroblasts transiently express transcription factors that are normally restricted to cells undergoing differentiation into glutamatergic neurons. This has led to the conclusion that some subtypes of glutamatergic OB neurons are produced throughout adult life [7]. The findings suggest that OB glutamatergic neurons are diverse in their origin. Gaining more insight into the molecular diversity of OB glutamatergic neurons could therefore help elucidate their precise function.

Transcription factors associated with postnatal glutamatergic OB neurogenesis include members of the basic helix-loop-helix family Neurod1 (ND1) and Neurogenin2 (Ngn2), and T-brain protein 1 (Tbr1) and T-brain protein 2 (Tbr2) [8]. ND1 is expressed in the SVZ by a subpopulation of OB progenitors [7, 9]. It is also expressed in cells along the entire RMS and is known to act during terminal differentiation of adult newborn OB neurons originating in the SVZ [7, 10]. The functional role of ND1during postnatal OB neurogenesis is not fully known [10, 11]. It is also unclear what phenotype migrating neuroblasts that express ND1 eventually adopt upon reaching the OB.

The primary objective of this study was to determine if OB glutamatergic neurons are developmentally diverse. Given that ND1 is commonly associated with cortical and hippocampal glutamatergic neurogenesis [12, 13], we hypothesized that ND1 expression is activated in the progenitor cells of multiple populations of OB glutamatergic neurons, including the mitral and tufted cells. We used genetic fate mapping and retroviral transgene delivery approaches to study the expression of ND1 during OB neurogenesis during the embryonic, postnatal and adult stages of neurogenesis in the rodent. We found the existence of several different populations of glutamatergic olfactory bulb neurons, the progenitors of which are ND1^+^ and ND1^-^ lineage-restricted, and are temporally and regionally separated. Our study brings new insights into the molecular diversity of OB glutamatergic neurons, which will help further elucidating their precise function.

## Materials and Methods

### Animals

*Neurod1* ^*tm1Jle*^ LacZ knock-in mice were previously described [12] and were bred and maintained on an outbred Black Swiss background (NTac:NIHBS, Taconic), according to Columbia University IACUC approved protocols. *Neurod1-cre* transgenic mice were generated by pronuclear injection of the *Neurod1-cre* BAC construct that carries cre-sequences downstream of the translational initiation codon ATG of the Neurod1 gene [14]. For cell fate mapping studies, *Neurod1-cre* transgenic mice were crossed with *ROSA26-EYFP* (B6.129X1-Gt (ROSA) 26Sortm1(EYFP) Cos) indicator mice [15]. Sprague dawley pregnant rats were ordered from B&K Universal Ltd, Sollentuna, Sweden. Animals were housed in groups with ad libitum access to food and water during 12 hours light:dark cycles. Animal experiments were approved by ethical committees at, and performed in accordance with the ethical guidelines of Lund University (approval number M233-06), Columbia University (approval number AAAC0259) and The New England Medical Center (approval number A1871-10), in accordance with the European Communities Council Directive of 24 November 1986 (86/609/EEC), and the NIH Guidelines.

### Tissue preparation

For immunohistochemical analysis, mouse and rat tissue was prepared as described thereafter. The heads from E15.5 embryos, newborn pups and 2 week-old mice were fixed in 4% paraformaldehyde (PFA) at 4°C overnight and then transferred into 30% sucrose/0.1 M phosphate buffer solution until sectioning on cryostat apparatus (10–14 μm thickness; Leica CM3000). Sections were mounted in series on Superfrost glass slides and stored at −80°C until immunohistochemistry was performed. Six week-old mice and two and six week-old rats were sacrificed by transcardial perfusion with saline for 5–10 minutes, followed by 4% PFA for 10 minutes. Brains were fixed and cryopreserved as described above until sectioning on a microtome apparatus (30 μm thickness sections, Microm Zeiss). Seven series of coronal sections were cut throughout the brain. Free-floating sections were preserved in anti-freeze solution until immunohistochemistry was performed.

### Virus production, in utero and in vivo surgeries

Infectious particles were produced using the producer cell line 293VSV-G [16], as previously described [17]. In utero experiments were conducted as previously described [18]. Briefly, 2 μl of viral suspension (titer = 1×10^^^9 TU/ml) was injected into the lateral ventricle of each embryo. Following normal delivery, the pups were allowed to develop up to two weeks. In vivo surgery was performed on 2 week-old rats. Animals were anesthetized with isoflurane, and viral particles (1.5x10^^^6 in 3 μl) were injected via stereotaxic surgery into the subventricular zone using the coordinates AP: +0.5; ML: -1; DV: -4.3, then raised up to -3.7 for injection (from Bregma). Animals were then removed from the stereotaxic frame and the wound cleaned and sutured. Marcaine (0.05ml) and analgesic were injected sub-cutaneously under the incision. Animals were also given 1 ml of warm sterile saline to prevent dehydratation, and placed in recovery unit. Procedures were carried out to minimize potential suffering of the animals. Animals were sacrificed 2 weeks (data not shown) and 4 weeks later and tissue was processed for immunohistochemistry.

### X-Gal staining

Tissue was stained overnight in the dark with X-Gal staining solution made by diluting 50 mg/ml X-Gal stock solution (dissolved in dimethylformamide) to a final concentration of 1 mg/ml in 0.1 M phosphate buffer containing 2 mM magnesium chloride, 5 mM potassium ferrocyanide (Sigma), and 5 mM potassium ferricyanide (Sigma). Slides were washed prior to coversliping.

### Immunohistochemistryand microscopy

Immunofluorescence was performed according to standard protocols. Tissue was fixed in 4% paraformaldehyde, rinsed with PBS three times prior to pre-incubation with a blocking solution (10% donkey serum, 0.25% Triton-X100 in PBS) for 1 hour. The remainder of the procedure was performed as previously described [18]. Specimen analyses were performed using Leica and Zeiss confocal microscopes, using Leica and Zen softwares, respectively. Samples were analyzed using 20×, 40× and 63× objectives, sometimes zoomed. Figures were composed in CANVAS-X software.

The antibodies used in this study are: goat anti-NeuroD1 (1:200; Santa Cruz), rabbit anti-Pax6 (1:150; Covance), rabbit anti-Tbr2 (1:500; Millipore), rabbit anti-Tbr1: (1:1000; Millipore), mouse anti-Nestin (1:500; R&D), mouse anti-NeuN (1:300; Millipore), rabbit anti-Calretinin (1:500; Swant). Rabbitanti-DARPP-32 (1:500, polyclonal, Millipore/Chemicon), rabbit anti-VGluT1 and rabbit anti-VGluT2 (1:1000,polyclonal, Synaptic System), mouse anti-Calbindin (1:1000, Sigma), rabbit anti-NPY (1:4000; Sigma), rabbit anti-GFAP (1:1000; DAKO), rabbit and mouse anti-tyrosine hydroxylase (Millipore and Pelfreeze), mouse anti-Parvalbumin (1:1000, Sigma), mouse anti-Meis1/2/3 (Millipore), Rabbit anti-SP8 (Millipore) and chicken anti-GFP (1:200, polyclonal, Millipore). We used a guinea pig anti-β-Galactosidase antibody (generous gift from Tom Finger’s laboratory, Colorado, to L.S.) and a chicken anti-β-Galactosidase (1:200, Millipore). The secondary antibodies Cy2, FITC, Cy3, and Cy5 (1:200) were purchased from Jackson IR laboratories (Suffolk, UK); Alexa-fluor-488, -568, and -647 (1:300) from Invitrogen/Molecular Probes (Paisley, UK). DAPI (1:1000) was purchased from Sigma.

### Quantification

Non-overlapping random field of views (4≤n≤10, depending on the extent to which GFP transduced cells could be identified), distant from each other by at least a third of a field of view, per section were counted using a 20x magnification objective. Between 7 and 10 sections from the same series per animal (with a periodicity of the section of 7), were counted and represent one experiment. No distinction was made with regards to the gradient of glomerular formation and mitral maturation known to occur during the first week of life [19, 20]. In the embryo, ND1-forced expression resulted in the formation of bilateral protrusions, which we referred to as ‘the bulges’. For the analysis of the bulge, the total number of GFP cells per series was counted and multiplied by the number of series to evaluate the total number of GFP cells. One animal or experiment represents one mean value and standard deviations were calculated between animals for n≥3 animals. All error bars are presented as standard errors of the mean (± s. e. m.). When relevant, the total number of cells counted in all animals is indicated in the figure legends.

## Results

### Expression of ND1 during OB development suggests a temporal and regional separation of ND1 progenitors

**Fig 1:**
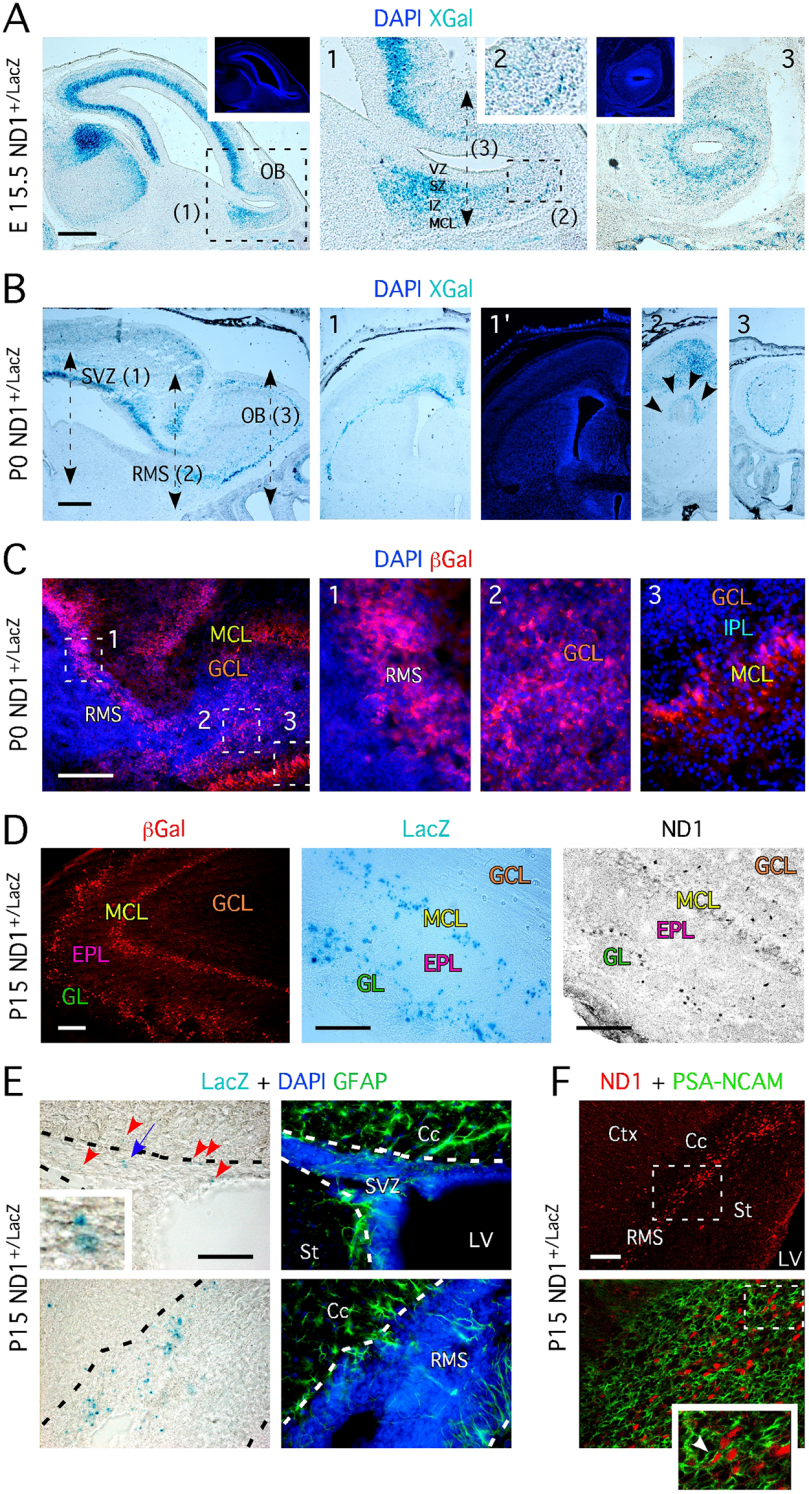
ND1 defines distinct populations of OB neurons born during the development and after birth. **A-** XGal staining on sagittal section of ND1^+/LacZ^ embryos aged E15.5 reveals the presence of β-Gal^+^ cells in the SZ, IZ and MCL of the forming OB. Insets show higher magnification of regions indicated by dashed frame. Panel 3 represents XGal staining performed on coronal section at the level of the black dashed double arrowed line. Insets located in the upper part show DAPI staining (in blue). **B-** XGal staining on sagittal section of ND1^+/LacZ^ newborn mice aged P0 shows the presence of β-Gal^+^ cells in the SVZ, the RMS, and the MCL of the OB. Insets (1, 2 and 3) show XGal staining on coronal sections at the level of each of these regions. DAPI staining of 1 is represented in 1’. In 2, multiple black arrowheads pinpoint at the outer and dorsal part of the RMS. **C-** Immunohistochemistry for β-Gal performed on sagittal section of ND1^+/LacZ^ mice aged P0 confirms the existence of ND1^+/LacZ^ cells revealed by XGal staining in B. Panels 1–3 represent higher magnifications of regions of interest marked by white dashed frames in the left panel. **D-** Numerous ND1 cells locate in the GL and MCL in the OB at P15, as revealed by XGal, β-Gal and ND1 stainings. **E-** ND1^+/LacZ^ cells locate in the SVZ and RMS of 2 week-old mice and are negative for the glial fibrillary acidic protein (GFAP). Red arrowheads pinpoint at XGal^+^ cells. Inset represents a higher magnification of XGal^+^ cells pinpointed by the blue arrowhead. Black dashed lines delineate the SVZ and RMS. **F-** Immunohistochemistry performed on sagittal section of 2-week old ND1^+/LacZ^ mice reveals that ND1^+^ are migrating PSA-NCAM^+^ neuroblasts. **A-**to **F-** Pictures are representative images of stainings perform on serial sections from n = 4–6 animals. VZ = ventricular zone, SZ = subependymal zone, SVZ = subventricular zone, IZ = intermediate zone, MCL = mitral cell layer, RMS = rostral migratory stream, GCL = granule cell layer, GL = glomeruli, IPL = internal plexiform layer, Cc = corpus callosum, St = striatum, LV = lateral ventricle, Ctx = cortex, OB = olfactory bulb. Scale bars: 200 μm (A-C) and 100 μm (D-F).

We investigated ND1 expression during OB development with the goal to identify putative glutamatergic progenitors. Using a knock-in mouse with LacZ in the ND1 locus (ND1^+/LacZ^) [12], we visualized ND1^+^ (via LacZ expression) cells in the olfactory bulb of E15.5 embryos, P0 and P15 pups. These periods of development represent the time during which the OB glutamatergic neuroblasts are born and the first glomeruli generated [3–5]. At E15.5, while the ventricular zone was devoid of XGal staining, we observed a strong concentration of XGal^+^ cells in the subependymal zone (SZ) throughout the forebrain (Fig 1A), and in the intermediate zone (IZ) and the developing mitral cell layer (MCL) of the bulb (Fig 1A1 & 1A2).

At birth, β-Gal^+^ cells were present in the dorsal SVZ lining the ventricular wall and dorsal RMS (Fig 1B), but not the lateral lining along the medial border of the striatum and the ventral part of the RMS (Fig 1B1 & 1B2), indicating region specific expression of ND1 in the SVZ. XGal staining in the olfactory bulb at birth was restricted to the MCL (Fig 1B3). This observation was confirmed using immunohistochemistry for β-Galactosidase (β-Gal; Fig 1C1–1C3). By two-weeks of age (P15), however, we noted a second population of XGal^+^ cells in the bulb, located in the glomerular layer (Fig 1D). This is in agreement with a previous report [10, 11], and we again confirmed the observation by immunohistochemistry for both β-Gal and ND1 (Fig 1D). It is worth mentioning that ND1 was not always identified in β-Gal^+^ cells due to its shorter persistence compare to the reporter.

Neurogenesis is known to contribute to the glomerular layer over time [21–23], but Boutin and coworkers [10] failed to see any ND1 RNA transcripts in the lateral ventricle and RMS of P5 brains. Given the postnatal appearance of the ND1^+^ cells in the glomerular layer, we questioned the origin of these cells. We examined LacZ and ND1 expression in the SVZ and RMS in P14 ND1^+/LacZ^ mice, and in agreement with previous reports [9, 11], we identified XGal^+^ cells in both the dorsal SVZ and the RMS, which suggests ND1^+^ cells continue to be produced at least two weeks after birth (Fig 1E). These cells also express PSA-NCAM in the RMS (Fig 1F), implying that they are migrating towards the OB, and may be the source of the ND1^+^ cells in the glomerular layer.

Based on these results, we hypothesized that there are two distinct populations of ND1-expressing cells during the early development of the OB, which are temporally and regionally separated. The first population appears to derive from the SZ of the developing OB and is destined to become mitral cells located in the MCL. The second population originates postnatally from the lining of the dorsal ventricular wall and migrates via the RMS to the glomeruli in the OB, ultimately becoming periglomerular neurons.

### Mitral cells express canonical glutamatergic markers during maturation

Mitral and tufted cells are excitatory OB neurons that originate from the OB SZ and are born during early embryonic development [24]. In contrast, periglomerular and granule inhibitory neurons originate from the SVZ lining the ventricular wall, and are born in late embryonic development and continuing throughout adult life [6, 25]. Our ND1 reporter mouse data demonstrate that ND1^+^ cells are present in these two distinct regions, suggesting that ND1^+^ progenitor cells could give rise to both excitatory and inhibitory neurons.

**Fig 2:**
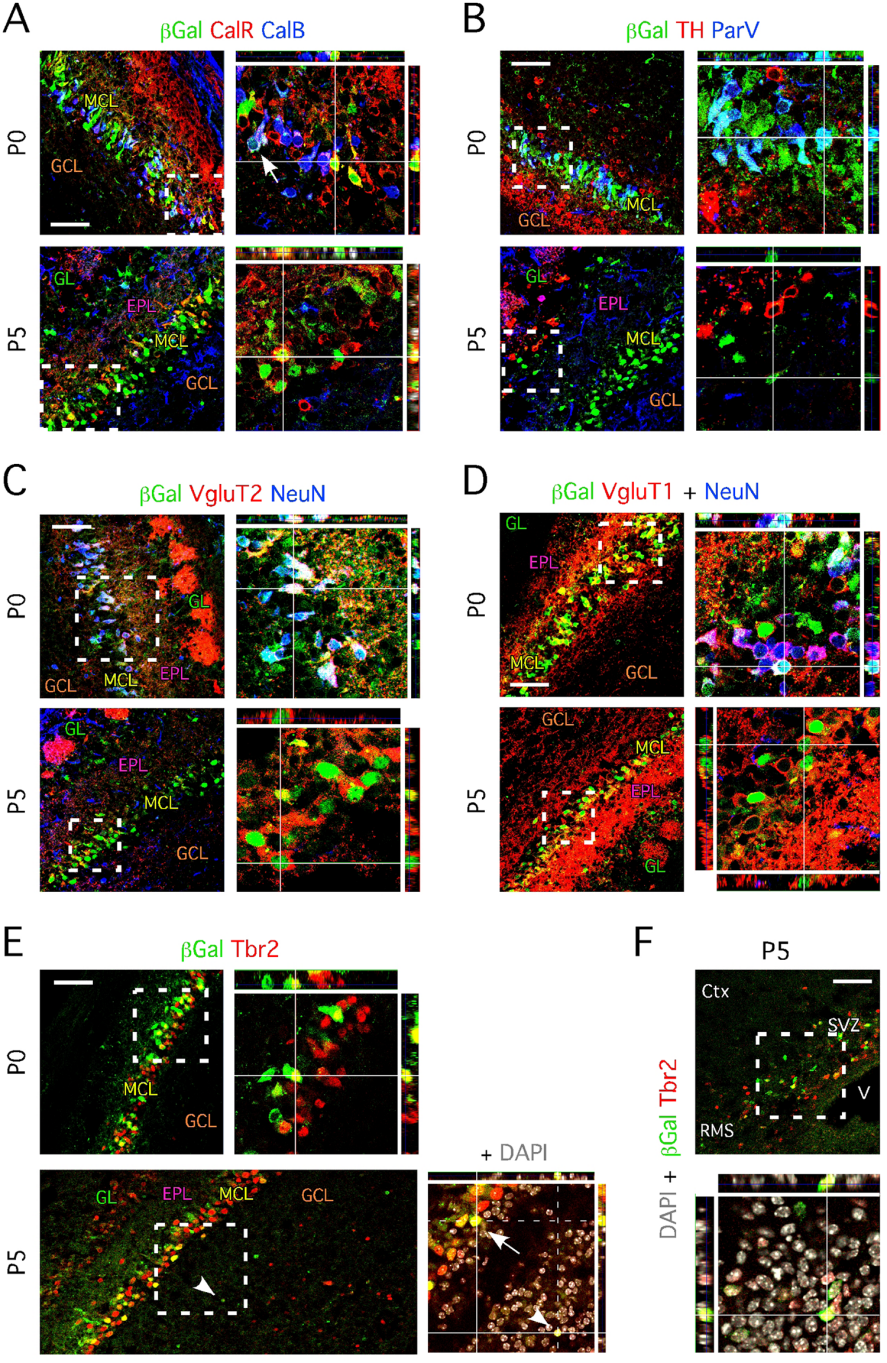
ND1-expressing cells located in the mitral cell layer transiently express markers associated with glutamatergic neuron phenotype. **A-** Immunohistochemistry of sagittal OB section of ND1^+/LacZ^ mice aged P0 shows β-Gal^+^ cells co-expressing CalR and CalB; white arrowhead pinpoint at a β-Gal^+^/CalR^+^/CalB^+^ triple labeled cell. β-Gal^+^/CalR^+^ cells are found in the mitral cell layer at P5; co-expression with CalB is lost at P5. Right panels represent higher magnification images of framed areas with orthogonal projections. **B-** Immunohistochemistry of sagittal OB section of ND1^+/LacZ^ mice aged P0 shows β-Gal^+^ cells co-expressing ParV. Co-expression with ParV is lost at P5. TH is never found co-expressed by β-Gal^+^ cells and only locates in β-Gal^-^ cells that migrate towards the forming glomeruli. Right panels represent higher magnification images of framed areas. **C-** Immunohistochemistry of sagittal OB section of ND1^+/LacZ^ mice aged P0 shows β-Gal^+^ cells co-expressing VGluT2 and NeuN. NeuN expression is lost at P5 while the expression of VGluT2 persists in β-Gal^+^ cells. Right panels represent higher magnification images of framed areas. **D-** Immunohistochemistry of sagittal OB section of ND1^+/LacZ^ mice aged P0 shows β-Gal^+^ cells co-expressing VGluT1 and NeuN. NeuN expression is lost at P5 while, as VGluT2, VGluT1 expression persists in β-Gal^+^ cells. Right panels represent higher magnification images of framed areas. **E-** Immunohistochemistry of sagittal OB section of ND1^+/LacZ^ mice aged P0 shows β-Gal^+^ cells co-expressing Tbr2. At P5, Tbr2^-^/β-Gal^+^, Tbr2^+^/β-Gal^-^ and Tbr2^+^/β-Gal^+^ (pinpointed by a white arrowheads) are observed in the granule layer and forming glomeruli. Right panels represent higher magnification images of framed areas and include DAPI staining (in grey). The white arrow pinpoints at a Tbr2^+^/β-Gal^+^ cell in the MCL. **F-** Tbr2^-^/β-Gal^+^, Tbr2^+^/β-Gal^-^ and Tbr2^+^/β-Gal^+^ locate in the SVZ of P5 animals. Lower panel represent a higher magnification image of the framed area and includes DAPI staining (in grey). **A-** to **F-**: SVZ = subventricular zone, MCL = mitral cell layer, RMS = rostral migratory stream, GCL = granule cell layer, GL = glomeruli, EPL = external plexiform layer, V = ventricle, Ctx = cortex. **A- to E-**: Images represent areas from middle sagittal sections of the anterior OB. Scale bars: 50 μm (A-F).

In order to address the nature of the cells in these populations, we first characterized ND1^+^ cells locating in the MCL by P0. We examined markers expressed in both excitatory and inhibitory neurons present in the different layers of the OB; these include parvalbumin (ParV), the enzyme responsible for catalyzing the conversion of the amino acid L-tyrosine to dihydroxyphenylalanine, expressed by GABAergic interneurons located in the external plexiform layer (EPL), tyrosine hydroxylase (TH) and the calcium-binding proteins calretinin (CalR) and calbindin (CalB) expressed by subpopulations of periglomerular inhibitory neurons [6, 18, 26]. At this time point, we determined that a third of β-Gal^+^ (ND1^+^) cells co-expressed either of the markers CalR (37 ± 2.6% [27], CalB (35 ± 5.5%) and ParV (32 ± 3.5%). In contrast at P5, β-Gal^+^ cells located in the MCL no longer expressed ParV, and only 18 ± 1.7% co-expressed CalB, whereas the majority co-expressed CalR (77 ± 3.2%). TH^+^/ β-Gal^+^ cells were never identified at P0 and P5 (Fig 2A and 2B). Interestingly, we identified few β-Gal^+^ cells surrounding the forming glomeruli; these did not express any of the markers studied, suggesting alternative fates for these ND1-expressing cells.

We next determined whether β-Gal^+^ cells located in the MCL were of the glutamatergic phenotype. We found that nearly all β-Gal^+^ cells express the vesicular glutamatergic transporters 1 and 2 (VgluT1 and VgluT2) [28] at both P0 and P5 (Fig 2C and 2D). However, at P5 we observed less intense expression of VGluT2 in the cell body and the projections of the β-Gal^+^ cells, while the expression of VGluT1 persisted (Fig 2D). This phenomenon was previously reported [29] and can now be ascribed to ND1-expressing cells. These data also confirm that β-Gal^+^ cells are not inhibitory GABAergic neurons. Moreover, β-Gal^+^ cells extend long VGluT2^+^ projections toward the forming glomeruli and transiently express the neuron nuclear antigen NeuN, which confirmed a mitral cell identity (Fig 2C and 2D) [30].

Thus, our data establish that the first population of OB cells expressing ND1 originate from the embryonic SZ, and become VGluT1/2^+^ glutamatergic mitral cells that transiently co-express CalB, ParV and CalR as they mature, similar to the phenomenon observed during hippocampal granule neurogenesis [8, 18, 31].

### Mitral cells express Tbr2 and segregate into ND1^+^ and ND1^-^ populations

The expression pattern of ND1 at P0 also appears to correspond with that of Tbr2 [32, 33]. Tbr2 is a member of the T-brain transcription factor family commonly associated with glutamatergic neurogenesis, specifically pyramidal neocorticogenesis and hippocampal neurogenesis [8]. Moreover, mice in which Tbr2 is knocked-down or conditionally deleted display atrophic OBs [34]. Given that ND1 and Tbr2 are part of the transcriptional cascade that elicits glutamatergic differentiation in both the neocortex and hippocampus, we hypothesized that Tbr2 is expressed in OB glutamatergic neurons that derive from ND1-expressing progenitors. At P0, nearly all (>99%) of the cells expressing β-Gal also expressed Tbr2 (Fig 2E), which represented 33.6 ± 3.8% of all Tbr2^+^ cells located in the mitral cell layer. This proportion was similar at P5 (35.7 ± 4.5%), suggesting that approximately one-third of all mitral cells are derived from cells that expressed ND1 in the early postnatal brain. Interestingly, at P5 we also found Tbr2^+^/β-Gal^+^ cells located in the granule layer, the internal plexiform layer, and surrounding the forming glomeruli (Fig 2E). Moreover, we found Tbr2^+^ cells in the SVZ, 26% of which co-expressed β-Gal (Fig 2F) and displayed a migration pattern typical of newborn neuroblasts migrating toward the OB (Fig 2F). This further supports the idea that Tbr2^+^/β-Gal^+^ cells define the second ND1-expressing population, which originates from the SVZ and migrate through the RMS, granule layer, mitral cell layer and external plexiform layer to reside in the forming glomerular layer.

### SVZ-derived ND1^+^ cells become periglomerular glutamatergic neurons

**Fig 3:**
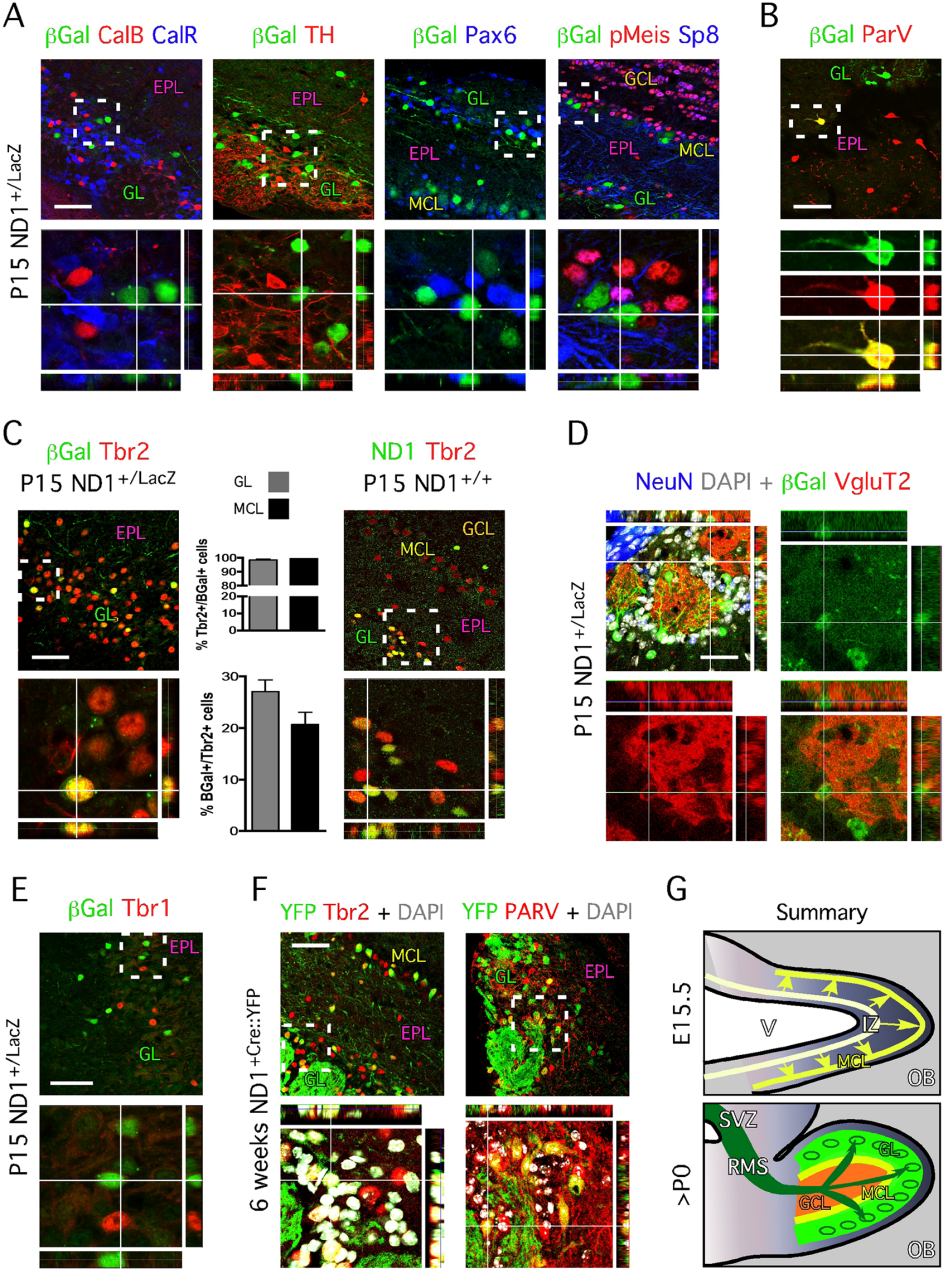
ND1 labels specific subset of neurons in the mitral and glomerular layers. **A-** Immunohistochemistry of sagittal OB section of 2-week old ND1^+/LacZ^ mice shows β-Gal^+^ cells do not co-express CalR, CalB, TH, Pax6, Meis and SP8 which are markers associated with GABAergic glomerular and periglomerular identities. Lower panels represent higher magnification images of framed areas. **B-** Rare β-Gal^+^/ParV^+^ neurons are identified in the external plexiform layer at P15. **C-** At 2 weeks of age, approximately a third of all Tbr2^+^ cells stain for β-Gal in the GL (27% ± 2.3%; 1942 cells counted in total) and MCL (20.6% ± 2.3%; 735 cells counted in total). Conversely, nearly all β-Gal^+^ cells co-express Tbr2 in the GL (98.3% ± 0.8%) and the MCL (99.3% ± 0.7%). Immunohistochemistry for ND1 on wildtype tissue confirms ND1 is specifically expressed in specific subset of Tbr2^+^ neurons in the mitral cell layer and glomeruli. **D-** β-Gal^+^ neurons are immuno-positive for the glutamatergic markers VGluT2. **E-** Tbr1^+^ neurons define subsets of OB glutamatergic neurons, which do not originate from a ND1 lineage. **F-** Permanent lineage tracing confirms the existence of both ND1-specific subtype diversity among Tbr2^+^ neurons and ParV^+^ neurons originating from ND1-positivelineage. **G-** Model summarizing origin, migration and location of ND1 progenitors and progenies at embryonic day E15.5 and postnatal stage. **A- to F-** Lower panels represent higher magnification images. SVZ = subventricular zone, MCL = mitral cell layer, RMS = rostral migratory stream, GCL = granule cell layer, GL = glomeruli, EPL = external plexiform layer, V = ventricle, OB = olfactory bulb. Values shown as mean ± s.e.m., *n* = 3 animals. Scale bars: 50 μm (A-F).

Our analysis identified Tbr2^+^/β-Gal^+^ cells in the granule cell layer and external plexiform layer, which are zones neurons must transit through in order to reach the forming glomeruli. To determine whether the ND1^+^ cells located in the forming glomeruli become inhibitory periglomerular neurons or glutamatergic periglomerular neurons, we performed immunohistochemistry on OB sections from both wild type and ND1^LacZ/+^ P15 animals. At this stage, β-Gal^+^ cells locating in the glomeruli did not co-express CalB, CalR, TH and Pax6 (Fig 3A), thereby excluding the possibility that SVZ-derived ND1^+^ cells had become periglomerular inhibitory neurons. Similar data were obtained using wildtype tissue and immunohistochemistry for ND1 and these markers (S1 Fig). In addition, these β-Gal^+^ cells lack expression of the GABAergic glomerular determinants Meis and SP8 (Fig 3A; [6], which further confirms that β-Gal^+^ cells are not GABAergic glomerular neurons. Interestingly, we identified few, if any (0–5 cells per OB section) β-Gal^+^ cells that co-expressed ParV (Fig 3B); these were not detected in wildtype tissue (S1 Fig), suggesting that ND1 expression is only transiently expressed in a minority of ParV^+^ neurons that locate in the glomeruli.

Similar to our observations at age P0 and P5, we observed that nearly all β-Gal^+^ cells located in the glomerular layer co-express Tbr2 at two weeks of age, which represents 27% of all Tbr2^+^ cells in this layer (Fig 3C). This was further confirmed with immunohistochemistry for ND1 and Tbr2 on wildtype OB sections from animals of similar age (Fig 3C). Consistent with our hypothesis, these data suggests that ND1 expression is maintained in approximately one third of Tbr2^+^ cells that originated from the SVZ and locate in the glomerular layer. To ascertain that ND1^+^ periglomerular neurons were glutamatergic neurons, we performed immunohistochemistry for β-Gal and the vesicular glutamate transporters. As expected, β-Gal^+^ cells co-expressed VgluT2 (Fig 3D) and VgluT1 (Data not shown), suggesting that the second ND1 expressing population originating from the SVZ also gives rise to glutamatergic neurons. Interestingly, we did not detect a single β-Gal^+^ cell co-expressing Tbr1 (Fig 3E), a marker employed to identify external tufted cells at postnatal stages [6], also shown to label a sub-population of juxtaglomerular neurons, possibly short axons cells [35]. Therefore this finding shows that Tbr1^+^ tufted cells may not belong to the ND1 lineage; however, we cannot exclude the possibility that both Tbr2^+^/ND1^-^ mitral cells and Tbr1^+^/ND1^-^ tufted cells originate from a common progenitor.

### Lineage tracing of ND1^+^ progenitors confirms the existence of a molecular diversity among mitral cells and glomerular neurons

It was still unclear whether there is a definitive segregation between Tbr2^+^/ND1^+^ cells and Tbr2^+^/ND1^-^ cells located in the mitral cell layer and the glomerular layer. It is possible that all mitral cells transiently express ND1, but only one-third maintained ND1 expression or expressed the reporter at the time of the analysis. We addressed this question by permanently marking the ND1 lineage using ND1-Cre mice [14] crossed with the Rosa26-YFP reporter mice (ND1-Cre/+^YFP/YFP^), and subsequently performed immunohistochemistry to identify co-expression of Tbr2 and YFP. Our findings were identical to those obtained using ND1^+/LacZ^ mice (Fig 3F), which demonstrated that one-third of Tbr2^+^ cells originates from a ND1 lineage. This proportion was similar for Tbr2^+^ cells located in the mitral cell and the glomeruli. In addition, we found YFP^+^ cells that co-express ParV, suggesting that a proportion of the ParV cells located in the glomerular layer also derive from a ND1 lineage and originate from the SVZ. These data confirmed our findings using ND1^+/LacZ^ mice (Fig 3B). As ParV expression in these cells is initially weak and diminishes over time [36], we could not determine the exact percentage of double-labeled cells, the majority of which we identified to be located in the ventral caudal region of the OB.

These results therefore suggest that a second population of ND1^+^ cells—co-expressing Tbr2—may originate from the dorsal SVZ. These cells would migrate from the SVZ to the OB and reside in the developing glomeruli ultimately adopting a glutamatergic fate (Fig 3G). Moreover, using transgenic mice that label ND1-derived or-expressing cells with YFP, we provide evidence for the existence of a third population of ND1^+^ cells that co-expresses ParV; more importantly, we confirm that both mitral cells and glutamatergic neurons located in the glomeruli originate from ND1^+^ and ND1^-^ lineages, thereby also suggesting that ND1 is not instrumental to glutamatergic identity.

### Glutamatergic OB neurons born postnatally originate from the SVZ are Tbr2-negative

**Fig 4:**
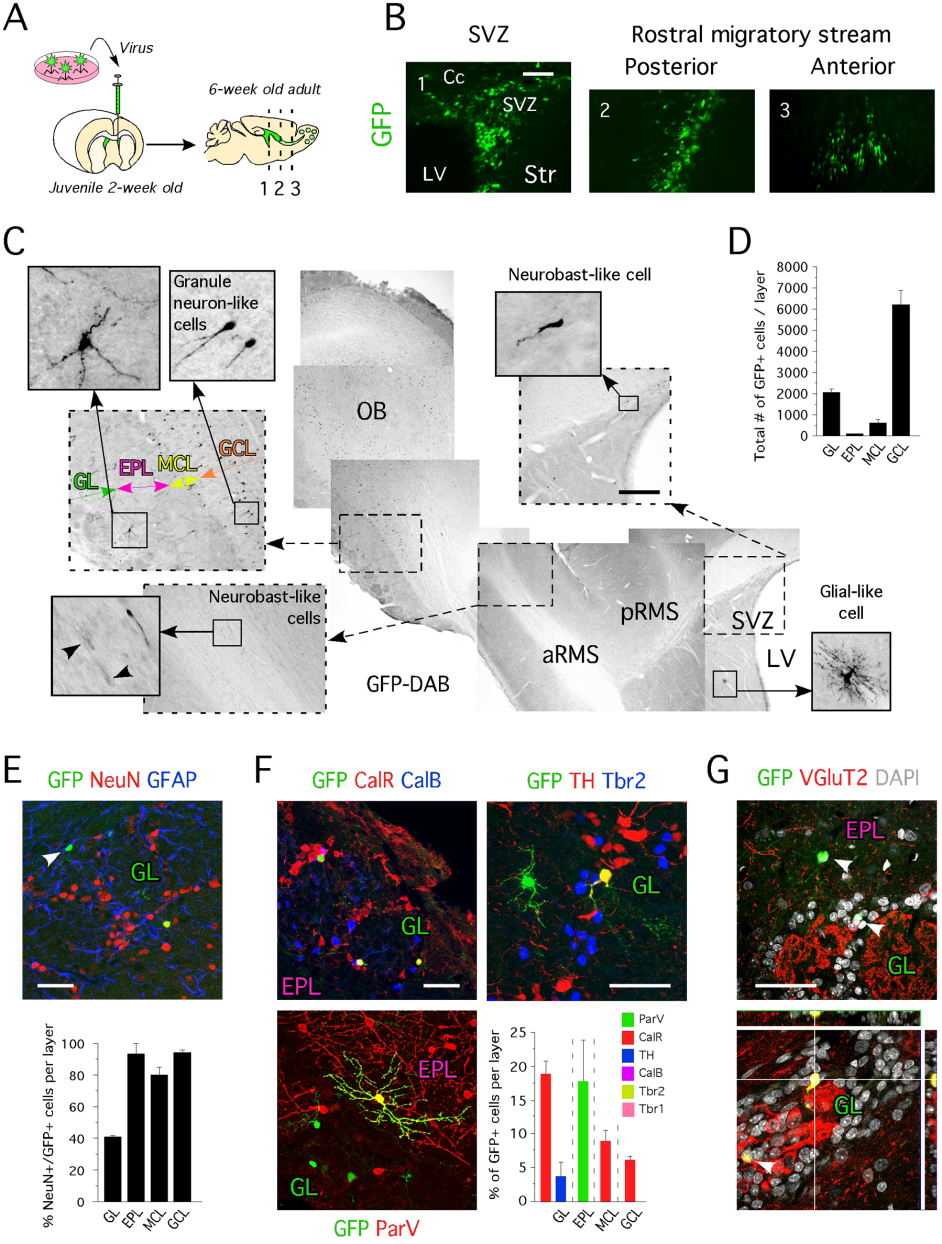
Identity of OB newborn neurons originating from the SVZ of 2 week-old animals. **A-** Cartoon illustrating the approach used for *in vivo* delivery of GFP, Ngn2 and ND1 in the SVZ of juvenile animals. **B-** Location of cells transduced with GFP virus 2 weeks post-injection revealed by immunohistochemistry for GFP. **C-** Location of cells transduced with GFP virus 4 weeks post-injection revealed by immunohistochemistry for GFP. **D-** Total number of GFP cells in specific layers of the OB (GL: 2070 ± 138 cells, EPL: 98 ± 12 cells, MCL: 621 ± 156 cells and GCL: 6242 ± 667 cells). **E-** Mature and immature GFP^+^ neurons can be identified in the glomerular layer. Percent of GFP^+^ cells expressing NeuN in the GL (40.7% ± 0.5%), the EPL (93.3% ± 6.6%), the MCL (80% ± 5%) and the GCL (94.1% ± 1.4%). **F-** Neuronal identity of GFP^+^ cells located in the GL, the EPL, the MCL and the GCL revealed by immunohistochemistry for ParV, CalR, TH, CalB, Tbr2 and Tbr1 (as % of GFP) shows absence of newborn TH^+^ dopaminergic and Tbr2^+^ and Tbr1^+^glutamatergic neurons in 2-week old injected animals. **G-** Immunohistochemistry for VGluT2 on sagittal section of 6-week old animals injected with GFP virus confirms the existence of VGluT2^-^ (upper panel) and VGluT2^+^ (lower panel) OB newborn neurons originating from the SVZ of 2-week old animals. **A to G-** Cc = corpus callosum, Str = striatum, SVZ = subventricular zone, MCL = mitral cell layer, pRMS = posterior rostral migratory stream, aRMS = anterior rostral migratory stream, GL = glomerular layer, EPL = external plexiform layer, MCL = mitral cell layer; GCL = granule cell layer, LV = lateral ventricle, OB = olfactory bulb. Values shown as mean ± s.e.m., *n* = 3 animals. Scale bars: 50 μm (B and E-G), 200 μm (C).

Previously, we demonstrated that OB progenitors transiently expressing Tbr2^+^ arise from the SVZ of 2-week old juvenile rodents [9]. In addition to recent findings [7, 35], these data suggest that an additional population of glutamatergic neurons that are Tbr2^-^/ND1^-^/VgluT2^+^ exist in the OB. Because forced-expression of ND1 in fate-committed neural progenitors leads to their exclusive differentiation into neurons, during the development and adulthood [9, 18, 37], we overexpressed ND1 by means of retrovirus (see experimental procedure) in SVZ progenitors of juvenile rats aged 2 weeks (Fig 4B), with the aim to trigger accelerated maturation, and seek GFP cells expressing VGluT2.

We identified the presence of GFP^+^ cells in all animals injected (n = 6) two weeks post-surgery; however, in contrast to animals injected with a control virus encoding just GFP (n = 6), or any other bHLH transcription factor tested (Ngn2 and ND2), the ND1^+^/GFP^+^ cells were only located in the SVZ and presented a neuron-like morphology (S2 Fig). This data confirms that ND1 accelerated the differentiation of juvenile SVZ progenitors since nearly all transduced cells immunostained positive for Doublecortin (S2 Fig). Unfortunately, at this stage, cells had not matured and therefore their cellular phenotype could not be assessed. After 4 weeks, no cells were detected in the SVZ of ND1 animals, implying the ND1 over-expressing cells could not form appropriate connections and died. At one month post-injection in control virus injected animal, only a few GFP^+^ cells, if any, were present in the SVZ and RMS (Fig 4C) and exhibited a neuroblast-like phenotype. The majority of GFP^+^ cells were located in the granule cell layers (approximately 6000 cells/animal), while 25% of the total population was located in the glomerular layer (Fig 4D). GFP^+^ cells were also located in the external plexiform layer and mitral cell layer. These observations were consistent among all animals injected.

**Fig 5:**
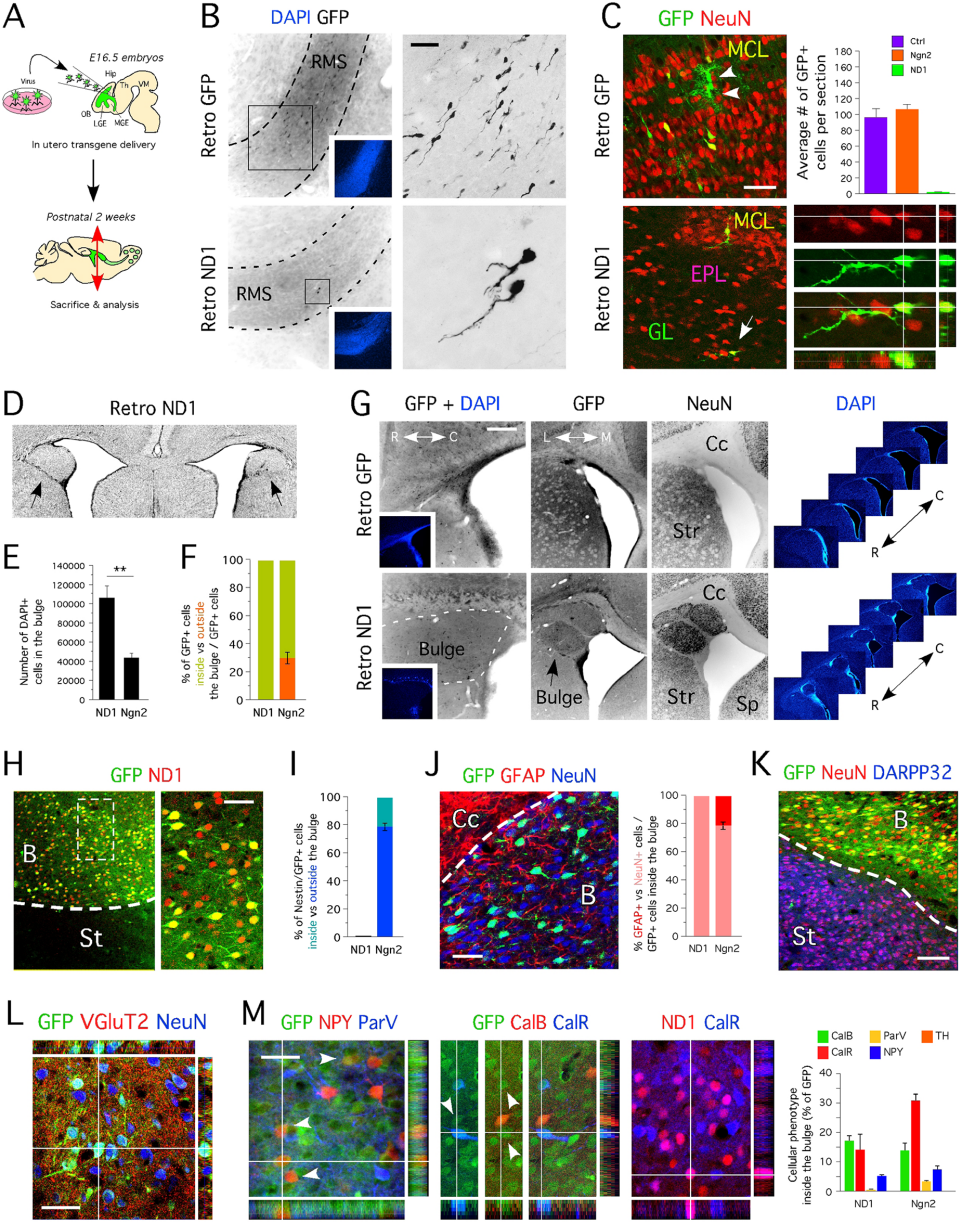
Forced expression of ND1 in at embryonic stage induces complete ectopic differentiation of OB neurons in the SVZ. **A-** Cartoon illustrating the approach used for *in utero* delivery of ND1, Ngn2 and GFP. **B-** Absence of cells transduced with ND1 retrovirus in the RMS 3 weeks post-injection revealed by immunohistochemistry for GFP. Right panels represent higher magnification images of areas framed on left panels. Insets show DAPI staining (in blue). **C-** Rare GFP^+^ cells are observed in the OB of animals injected with ND1 retrovirus compare to animals injected with Ngn2 (107 ± 3.1 cells) and control retrovirus (97 ± 6.4 cells). **D-** Bilateral protrusions in the SVZ revealed by DAPI staining on coronal sections of a 2-week old animal injected with ND1 retrovirus. Black arrowheads pinpoint at the bulges. **E-** Total number of cells in the bulge of animals injected with ND1 (106453 ± 11715 cells) and animals injected with Ngn2 (43621 ± 4566 cells) retroviruses. **F-** Absence of ND1 transduced cells outside the bulge revealed by immunohistochemistry for GFP. **G-** Anatomical structure of the bulge revealed by immunohistochemistry for GFP and NeuN on sagittal and coronal sections; DAPI staining (in blue) on serial coronal sections shows the bulge specifically locates in the SVZ region. **H-** Transduced cells located in the bulge express ND1 and GFP. **I-** Absence of nestin^+^/GFP^+^ cells in the bulge of ND1 transduced animals indicates transduced SVZ progenitors have matured. **J-** Nearly all SVZ neural progenitors tranduced with ND1 virus (99.1% ± 0.3%) become NeuN^+^ neurons. SVZ neural progenitors tranduced with Ngn2 virus become NeuN^+^ neurons (78.6% ± 2.3%) and GFAP^+^ astrocytes (22.4% ± 2.5%). **K-** None of the SVZ neural progenitors transduced with ND1 (and Ngn2; data not shown) become DARPP32^+^ striatal medium spiny neurons. **L-** Neurites of ND1-transduced neurons (bright GFP^+^ cells) stain immune-positive for VGluT2. **M-** SVZ neural progenitors transduced with ND1 differentiate into NPY^+^ (7.4% ± 1.2%), ParV^+^ (3.4% ± 0.3%), CalR^+^ (30.9% ± 2%) and CalB^+^ (13.9% ± 2.4%) neurons. Forced expression of ND1 in SVZ progenitors accelerate neuronal differentiation without modifying neuronal identity (here shown for CalR neurons). Like ND1, Ngn2 did not modify neuronal identity. **A to M-** Hip = hippocampus, VM = ventral mesencephalon, MGE = medial ganglionic eminence, Cc = corpus callosum, Str = striatum, Sp = septum, B = bulge, SVZ = subventricular zone, MCL = mitral cell layer, RMS = rostral migratory stream, GL = glomeruli, EPL = external plexiform layer, V = ventricle, OB = olfactory bulb. Values shown as mean ± s.e.m., *n* = 3 animals. Scale bars: 25 μm (H, J, K and M), 50 μm (K, B and C) and 100 μm (G).

We next assessed the phenotype of GFP^+^ cells and found GFP^+^ cells co-expressing CalR, TH and ParV (Fig 4E), but never CalB, Tbr1 or Tbr2, which confirms previous reports that OB neurons that maintain Tbr2 expression are found only a few days after birth and not at 2 weeks of age [7]. Moreover, we identified NeuN^-^ cells with a neuronal-like morphology, and immunohistochemistry confirmed that these were not glial cells (Fig 5F), suggesting that these cells could be immature neurons. Importantly, we also identified newborn glutamatergic neurons GFP^+^/VGluT2^+^. We identified 21% of GFP^+^ cells located in the glomerular layer to co-express VGluT2 (Fig 4G) [38]. Together, these data support the previous observations that an additional population of glutamatergic neurons exists, the progenitors of which also reside in the SVZ and migrate via the RMS to the OB [7, 9].

### Ectopic over-expression of ND1 in utero reveals the fate of SVZ progenitors

The forced expression of ND1 was not informative when performed in juvenile animals because a that stage, survival of neurons in an ectopic location appeared suboptimal, as previously suggested [39]. Therefore, we thought to trigger maturation of progenitors located in the SVZ, at earlier time point. Using intraventricular delivery, we injected ND1 retroviruses into E15.5–16.5 rat embryos and examined the phenotype of the transduced cells in juvenile animals (Fig 5A). We chose to perform viral injections in rats because these animals share a ND1 expression pattern similar to the mouse [40], and the size of rat embryos *in utero* allowed for efficient delivery [18].

Even when injected during the development, ND1 abolished the migration of transduced SVZ progenitors (Fig 5B). As a consequence, as few as 2–4 GFP^+^ cells were detected per OB section of ND1 injected rats, while control rats had approximately 30 times more GFP^+^ cells per section (Fig 5C). Since the ND1-overexpressing cells were not present in the RMS or the OB, we examined the SVZ of ND1 injected animals and discovered that the majority, if not all, of the cells transduced with ND1 had remained in the SVZ. ND1-expressing cells formed bilateral protrusions, which we arbitrarily describe as ‘the bulges’ (Fig 5D). All animals injected with ND1 virus (n = 6) exhibited a similar phenotype. Conversely, none of the animals injected with the control virus (n = 6) displayed the bulges, suggesting that this cellular structure was not an artifact of the injection procedure or the presence of virus itself. Moreover, all ND1-transduced cells exhibited a neuronal-like morphology, as confirmed by NeuN staining (Fig 5I), which suggested that ND1 had triggered exclusive neuronal differentiation of transduced SVZ progenitors. To confirm the unique ability of ND1 to direct neuronal maturation in vivo, we also examined the effect of the bHLH transcription factor Ngn2. Similar to animals injected with the control virus, GFP^+^ neuroblasts were found in the RMS of Ngn2 injected animals (data not shown), and as a result a similar magnitude of GFP^+^ cells was identified in the OB in these animals (Fig 5C). Interestingly, we observed that Ngn2 injected animals (n = 5) also contained a bulge located on either side of the brain in the SVZ (data not shown); however smaller in size to that of ND1 injected animals (Fig 5E). This confirmed the limited capability of Ngn2 transcription factor to also trigger exclusive neuronal commitment in vivo [18].

Since no bulge was generated following injection of the control virus, we performed a comparative analysis of the Ngn2-injected animals with the ND1-injected animals in order to characterize the cells present in the bulges. While all GFP^+^ cells were identified in the bulge of ND1 injected animals, 25% of those present in Ngn2 animals were externally located and had maintained the ability to migrate towards the OB (data not shown). All GFP^+^ cells located in the bulge of ND1 injected animals expressed the transgene (Fig 5H) and all developed into neurons (Fig 5J). In contrast, 20% of GFP^+^ cells in the bulge of Ngn2 animals matured into glial cells, and many still expressed the neural progenitor marker nestin (Fig 5I and 5J). This result supports our previous observation that the effect of ND1 on neuronal induction is greater than that of Ngn2 [18], and reinforces the role of Ngn2 in the maintenance of a neural progenitor state [41].

None of the GFP^+^ cells locating in the bulges of Ngn2 and ND1 injected animals, expressed DARPP32 marker (Fig 5K). This result implies that none of the SVZ transduced neural progenitors differentiated into medium spiny neurons. Within the bulge, we identified that the majority of OB neuronal subtypes including GFP^+^ neurons strongly labeled for VGluT2^+^ at the axonal and dendritic levels, in both animals injected with ND1 (Fig 5L) and those injected with Ngn2 (data not shown). These data indicate that OB glutamatergic progenitors had matured ectopically in the SVZ, and authenticate the existence of glutamatergic OB progenitors in the embryonic SVZ.

## Discussion

**Fig 6:**
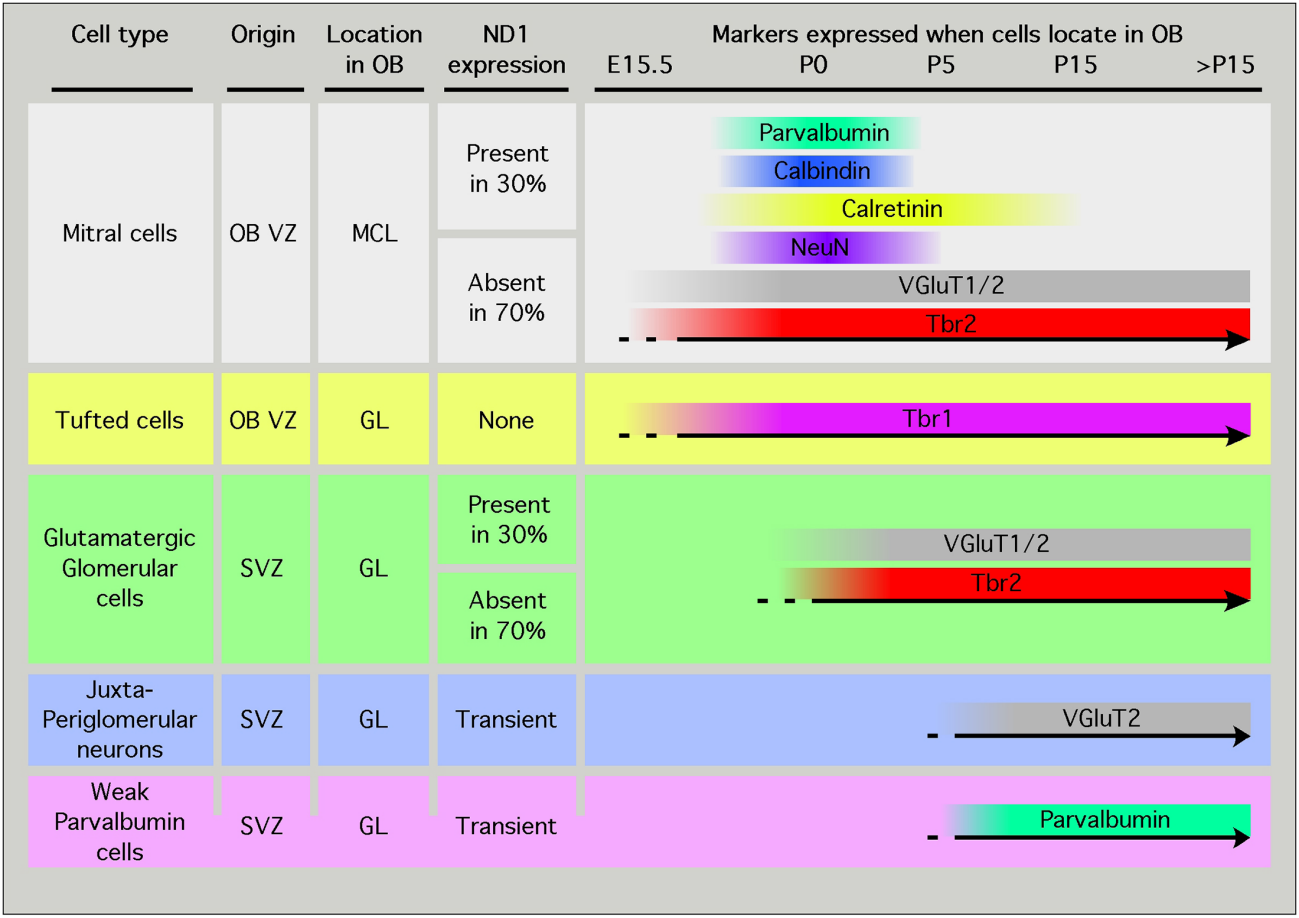
Summary of OB neuron subtypes highlighted in this study. Based on ND1 lineage tracing studies, we identified subset populations within the mitral and glutamatergic glomerular neuronal pool. In each of these populations, a third of the cells originate from a ND1 lineage. Tufted cells expressing Tbr1 originate from a ND1 negative lineage. Permanent lineage tracing study in ND1-Cre/+^YFP/YFP^ animals confirm the data obtained using a knock-in mouse model of LacZ into the ND1 locus, and reveals the existence of weakly-expressing parvalbumin cells that originate from a ND1 lineage. In vivo labeling of SVZ progenitors reveals the existence of ND1 negative—VGluT2 positive OB neurons born after birth.

The objective of this study was to determine the diversity of OB glutamatergic neurons. We used ND1 knock-in and transgenic animals to identify and lineage-trace the fate of putative glutamatergic progenitors. We demonstrate the existence of neuronal subgroups within the mitral cells (Tbr2^+^/ND1^+^ and Tbr2^+^/ND1^-^), within the periglomerular Parvalbumin^+^ cells (ParV^+^/ND1^+^ and ParV^+^/ND1^-^) and within the glutamatergic juxtaglomerular neuronal population (Tbr2^+^/ND1^+^, Tbr2^+^/ND1^-^ and Tbr2^-^/ND1^-^). This diversity (Fig 6) has not been previously reported at the molecular level, although it likely reflects the previously documented functional and morphological heterogeneity associated with these populations. Moreover, we demonstrate that Tbr1^+^ tufted cells originate from a ND1^-^ lineage.

### Molecular diversity within the mitral cell pool revealed by ND1

The question of whether diversity exists among mitral cells has been addressed using 5-bromo-2'-deoxyuridine (BrdU)-birth dating experiments and clearly demonstrated that the timing of neurogenesis of BrdU-labeled mitral cells influences on their location [24]. This suggested the existence of a positional diversity, which may potentially impact on the functional specificity of the different zones of the odorant receptor map. Coupled to dye injection, this method becomes powerful and allows one to identify input connections with associated cortical areas. We have now demonstrated the existence of a molecular diversity among mitral cells. The analysis of the OB of ND1^+/LacZ^ knock in mice at P0 and P15 revealed that a third of Tbr2^+^ mitral cells originate from a ND1-positive lineage. The subsequent lineage trace of ND1 cells (ND1-Cre/+^YFP/YFP^) confirmed that ND1 expression is not transient, but rather is maintained in one third of all mitral cells. The function of these distinct populations of mitral cells will need further investigation. It is worth mentioning that incomplete recombination may occur when using Cre driver animals; however, since similar proportions of ND1-expressing OB glutamatergic neurons were identified using the two models, incomplete recombination seemed unlikely.

### Molecular diversity within the tufted cell pool revealed by ND1

Olfactory glomeruli are formed during late development [42]. A heterogeneous population of juxtaglomerular neurons that includes periglomerular, short axon, and external tufted cells surrounds OB glomeruli. We observed ND1 expression overlapping with that of Tbr2, but not Tbr1 in the glomerular layer. This strongly suggests that different types of external tufted cells exist.

So far, measures of electrophysiological and morphological properties have been used to demonstrate the existence of two distinct subpopulations of external tufted cells [43]. We found that similar to the mitral cells, a third of all Tbr2^+^ tufted cells co-express ND1. It is suggested that ND1 plays a critical role in dendrite growth as well as branching development [44]. In agreement with this, adult newborn granule neurons in the hippocampus in ND1 null mice display shorter dendrites compared with wild-type neurons [11]. Therefore it is possible that ND1^+^/Tbr2^+^ cells represent the third of external tufted cells previously identified by Antal and coworkers, and described as external tufted cell “cluster 2” [43].

### Extrabulbar origin of glutamatergic neurons revealed by ND1

Postnatally, we identified Tbr2^+^ cells in the SVZ, and a third co-expressed β-Gal and displayed a migration pattern typical of newborn neuroblasts migrating towards the OB. Not only is this finding evidence for the existence of an additional population of Tbr2^+^/β-Gal^+^ cells (in addition to Tbr2^+^/β-Gal^+^ mitral cells), it confirms the extrabulbar origin of some of the glutamatergic juxtaglomerular neurons [35]. In this study, we identified an additional population using retroviral labeling. We demonstrated that, upon reaching the OB, these neurons did not express Tbr2, something we previously identified in wildtype juvenile rodent [9], and was identified by others as glutamatergic olfactory bulb interneurons produced throughout life [7].

### ND1 is a marker of diversity for the generic glutamatergic transcriptional code

ND1 is part of the transcriptional cascade elicited during cortical and hippocampal glutamatergic neurogenesis [8]. From the characterized temporal expression of transcription factors associated with glutamatergic differentiation in these areas, ND1 expression was shown to begin after that of Ngn2 and Tbr2, but before that of Tbr1. Moreover, the forced expression of ND1 reveals that this transcription factor is sufficient to induced Tbr1 expression in progenitors derived from these areas [9, 37, 45].

Mitral and tufted cells share common origins and segregate based on the maintenance of Tbr1. Immunohistochemical analysis performed on OB sections from tamoxifen-induced Ngn2-Cre^ERT2^;Tau^GFP^ animals indeed revealed that as early as E13.5, Ngn2 progenitors give rise to Tbr2 and Tbr1 co-expressing cells derived from the OB VZ [35]. Thus, Tbr1 expression appears to be lost once the cells establish in the mitral layer. Importantly, our data demonstrate that not all mitral cells and periglomerular glutamatergic neurons follow the generic code of Ngn2 → Tbr2 → ND1 → Tbr1. Approximately two thirds of these cells do not express ND1, which further confirms that ND1 is neither required for glutamatergic identity nor, as previously suggested, required for terminal differentiation of all SVZ progenitors [10], but rather of specific neuronal subpopulations originating from both the SVZ and OB.

Our data also suggest that mitral cells transiently co-express NeuN, CalB, ParV and CalR as they mature. To our knowledge this is the first report of the co-expression of these markers by mitral cells. This resembles observations made during hippocampal granule neurogenesis, with the exclusion of ParV expression [8, 31]. Whether CalB, ParV and CalR are expressed in the same sub-lineage or define a secondary subgroup within the ND1-expressing mitral cell pool needs further investigation.

### ND1 promotes exclusive neuronal induction of fate-committed SVZ progenitors

In the embryo, ND1-forced expression resulted in the formation of bilateral protrusions, which we referred to as ‘the bulges’. All animals injected with ND1 virus exhibited a similar phenotype. When ND1 expression was forced postnatally, we identified the presence of GFP^+^ cells in all animals injected two weeks post-surgery; however, in contrast to animals injected with a control virus or the bHLH transcription factor Ngn2 used as control gene, only ND1^+^/GFP^+^ cells were located in the SVZ. This data confirms that ND1 accelerated the differentiation of the SVZ progenitors and arrested both migration and differentiation such that Doublecortin^+^ neurons were produced exclusively; no cells matured and therefore the phenotype of these cells could not be assessed. No GFP^+^ cells could be detected in the SVZ of ND1 transfected animals four weeks after surgery, implying the ND1 over-expressing cells could not form appropriate connections and died. Importantly, we confirmed that glutamatergic progenitors reside in the SVZ and that ND1 is sufficient to instruct generic neuronal differentiation without changing neuronal identity, in concurrence with our previous findings *in vitro* [9].

### ND1 reporter mice as a new tool to study molecular diversity and mitral cell functionality

ND1 reporter mice represent a useful tool by which many unresolved issues can be addressed. For example, they can be used to directly visualize ND1^+^ mitral cell projections to the olfactory cortex using 3D reconstruction, and allow for the characterization of specific cortical areas targeted by these cells. The use of ND1-driven fluorescent reporter animals and laser-capture microscopy will make it possible to specifically isolate the two different mitral cell types and define how they differ at the molecular level. Studies using conditional genetic knock down could be performed to address how selective loss of mitral and tufted cells, affect functional processing of sensory information in the OB [46].

## Supporting Information

S1 Fig ND1 is absent in labels specific subset of neurons in the mitral and glomerular layers. **A-** Immunohistochemistry of sagittal OB section of 2-week old WT mice shows that ND1^+^ cells do not co-express CalR, CalB, TH and Pax6 markers associated with GABAergic glomerular and periglomerular identities. **B-** Immunohistochemistry of sagittal OB section of 2-week old WT mice shows absence of ND1 in ParV, Tbr1 and Meis and Sp8 expressing cells. **A- and B-** Lower panels represent higher magnification images. GL = glomeruli, EPL = external plexiform layer. Images are representative of *n* = 3 animals. Scale bars: 10 μm (M), 25 μm (H, J and K), 50 μm (K, B and C) and 100 μm (G).
(TIF)

S2 Fig ND1 transduced SVZ progenitors do not migrate via the RMS, and mature in the SVZ. **A-** Location of cells transduced with GFP, ND1, Ngn2 and NeuroD2 (ND2) virus, 2 weeks post-injection revealed by immunohistochemistry for GFP. Almost no GFP^+^ migrating cells can be identified in the RMS of ND1 injected animals. **B-** GFP+ cells transduced with ND1 are post-mitotic neuroblasts expressing DCX. Immunohistochemistry for KI67 reveals that GFP+ cells transduced with the control virus are still mitotically active, as opposed to ND1 transduced cells. Scale bars: 50 μm (A and B).
(TIF)
