# Supplementary figures and images for: The Origin, Development and Molecular Diversity of Rodent Olfactory Bulb Glutamatergic Neurons Distinguished by Expression of Transcription Factor NeuroD1
Source: PLoS One. 2015 Jun 1;10(6):e0128035. doi: 10.1371/journal.pone.0128035 (PMC4451148; doi:10.1371/journal.pone.0128035)

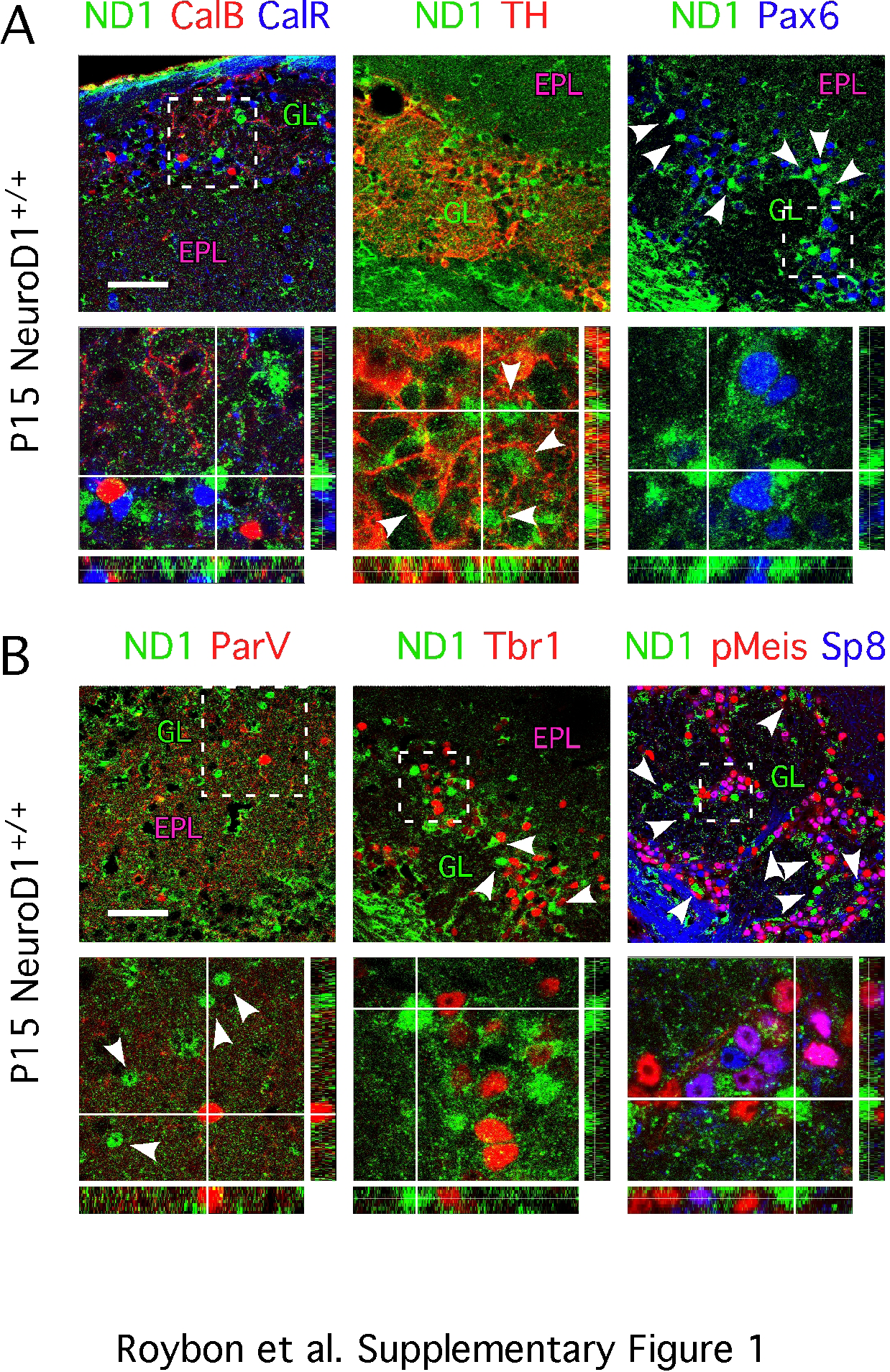

Supplement: S1 Fig — A- Immunohistochemistry of sagittal OB section of 2-week old WT mice shows that ND1+ cells do not co-express CalR, CalB, TH and Pax6 markers associated with GABAergic glomerular and periglomerular identities. B- Immunohistochemistry of sagittal OB section of 2-week old WT mice shows absence of ND1 in ParV, Tbr1 and Meis and Sp8 expressing cells. A- and B- Lower panels represent higher magnification images. GL = glomeruli, EPL = external plexiform layer. Images are representative of n = 3 animals. Scale bars: 10 μm (M), 25 μm (H, J and K), 50 μm (K, B and C) and 100 μm (G). (TIF) [file pone.0128035.s001.tif]

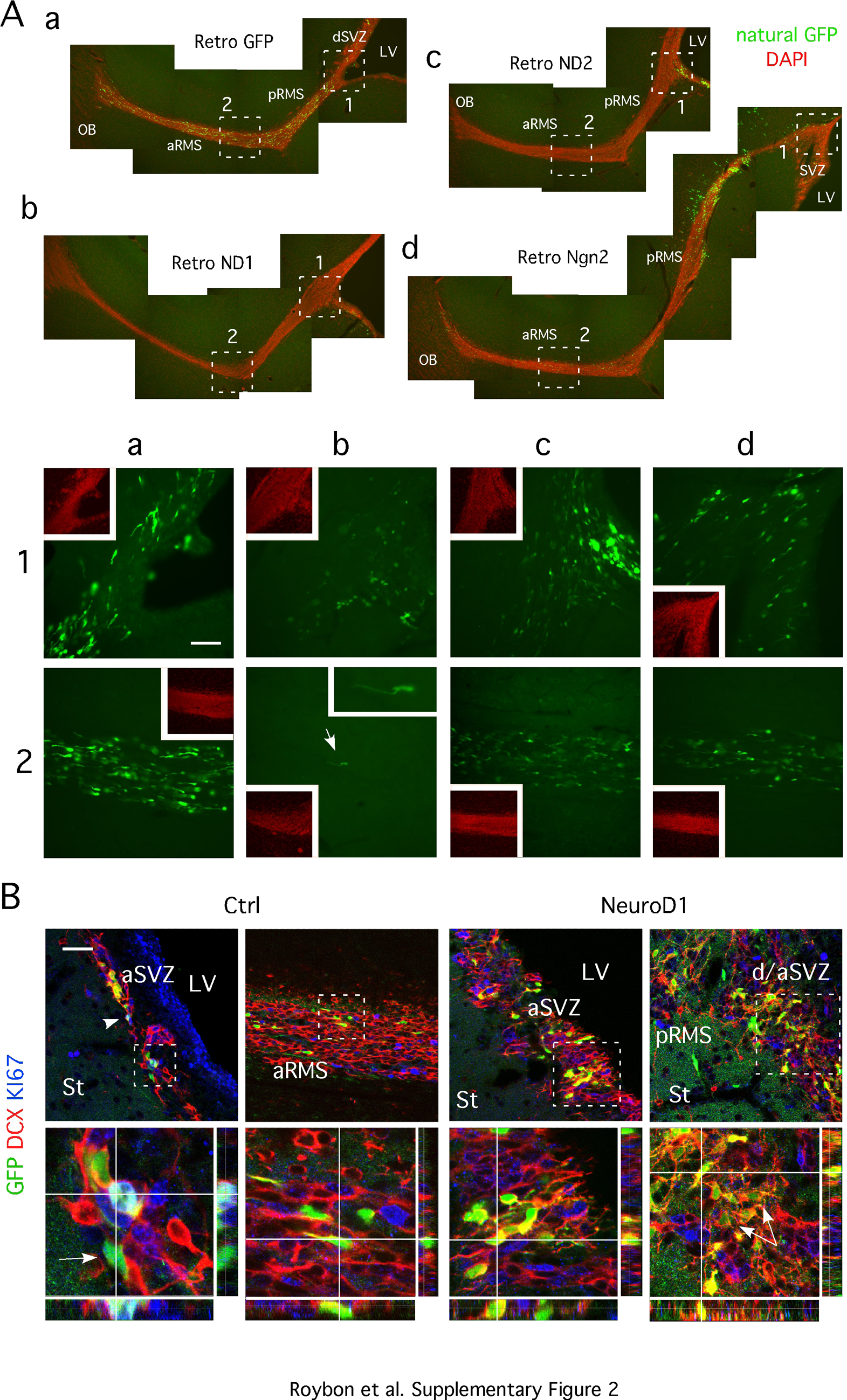

Supplement: S2 Fig — A- Location of cells transduced with GFP, ND1, Ngn2 and NeuroD2 (ND2) virus, 2 weeks post-injection revealed by immunohistochemistry for GFP. Almost no GFP+ migrating cells can be identified in the RMS of ND1 injected animals. B- GFP+ cells transduced with ND1 are post-mitotic neuroblasts expressing DCX. Immunohistochemistry for KI67 reveals that GFP+ cells transduced with the control virus are still mitotically active, as opposed to ND1 transduced cells. Scale bars: 50 μm (A and B). (TIF) [file pone.0128035.s002.tif]
